# Increased Long-Flight Activity Triggered in Beet Armyworm by Larval Feeding on Diet Containing Cry1Ac Protoxin

**DOI:** 10.1371/journal.pone.0063554

**Published:** 2013-05-13

**Authors:** Xing Fu Jiang, Jian Chen, Lei Zhang, Thomas W. Sappington, Li Zhi Luo

**Affiliations:** 1 State Key Laboratory for Biology of Plant Diseases and Insect Pests, Institute of Plant Protection, Chinese Academy of Agricultural Sciences, Beijing, China; 2 United States Department of Agriculture - Agricultural Research Service, Corn Insects and Crop Genetics Research Unit, Genetics Laboratory ISU, Ames, Iowa, United States of America; University of Tennessee, United States of America

## Abstract

Evaluating ecological safety and conducting pest risk analysis for transgenic crops are vitally important before their commercial planting. The beet armyworm, *Spodoptera exigua,* a long-distance migratory insect pest, is not a direct target of transgenic Cry1Ac-expressing cotton in China, but nevertheless it has recently become an important pest. Migrants leaving their natal field arrive in other appropriate habitat far away in a short time, often followed by larval outbreaks. *S. exigua* has low susceptibility to Cry1Ac. However, our results from laboratory experiments identified (i) sublethal effects of Cry1Ac protoxin on larval development rate, larval and pupal weight, and adult lifetime fecundity, and (ii) increased long-flight behavior triggered by Cry1Ac which may contribute to larval outbreaks elsewhere. No significant differences in larval mortality, pupation rate, adult emergence rate, longevity, pre-oviposition period, or oviposition period were observed between controls and larvae fed on artificial diet incorporating a low concentration of Cry1Ac protoxin. The negative sublethal effects on some developmental and reproductive traits and lack of effect on others suggest they do not contribute to the observed severity of *S. exigua* outbreaks after feeding on Cry1Ac cotton. Interestingly, the percentage of long fliers increased significantly when larvae were reared on diet containing either of two low-dose treatments of Cry1Ac, suggesting a possible increased propensity to disperse long distances triggered by Cry1Ac. We hypothesize that negative effects on development and reproduction caused by Cry1Ac in the diet are offset by increased flight propensity triggered by the poor food conditions, thereby improving the chances of escaping adverse local conditions before oviposition. Increased long-flight propensity in turn may amplify the area damaged by outbreak populations. This phenomenon might be common in other migratory insect pests receiving sublethal doses of Bt toxins and warrants further study.

## Introduction

Transgenic crops that produce toxins from *Bacillus thuringiensis* (Bt) for insect control have become an important component of pest management worldwide [1]. Bt crops can significantly suppress the target pest population, resulting in a reduction of chemical pesticide applications and increased crop productivity. Because of their compatibility with the environment, they are being approved for planting in more and more countries [2], [3]. In China, the main Bt crop is cotton, with a total of 2.4 million ha planted since its commercialization in 1997, resulting in large-scale insecticide reduction [4]. Bt cotton expressing the Cry1Ac toxin has been planted in northern and eastern China especially to control the target pest *Helicoverpa armigera* [5]. However, this widespread planting of Bt cotton has been accompanied by concerns of its sustainability and ecological safety. For example, concerns include the risk of resistance developing in the target pests, and release or evolution of secondary non-target pests. In fact, with the widespread adoption of Bt cotton in China, several non-target insect pests, such as mirid bugs [6], aphids [7], and *Spodoptera* spp. armyworms [8]–[10], including the beet armyworm, *Spodoptera exigua*, have become major pests in Bt cotton fields. Therefore, characterizing Bt toxicity to non-target pests and their effects on population dynamics have become an important avenue of research in recent years.

Transgenic Bt cotton expressing the Cry1Ac toxin was introduced commercially in North America in 1996, and in China in 1997. It is very effective against target lepidopteran pests such as tobacco budworm, *Heliothis virescens*, and pink bollworm, *Pectinophora gossypiella*. In China a key target pest is the cotton bollworm, *H. armigera*, but it is somewhat tolerant of the Cry1Ac toxin [11], as is its sister species, *H. zea*, in North America [12]. *S. exigua* is even more tolerant to Cry1Ac [13]–[18] and can infest Bt cotton expressing this single toxin [15], [19], [20]. *S. exigua* has become a serious economic pest of agriculture across China, infesting a wide geographic area through annual long distance migration [21]–[23]. Since Bt cotton varieties, mostly expressing the Cry1Ac toxin, were first planted commercially in China, *S. exigua* populations in Bt cotton fields have not been suppressed, but rather have increased [24]–[27]. Likewise, in Pakistan, Bt cotton expressing Cry1Ac is not effective in controlling *S. exigua* infestations [28].

This raises important questions about whether the natural tolerance of *S. exigua* to Bt crops expressing Cry1Ac [15], [17] increases its risk as a pest. No evidence suggests that *S. exigua* outbreaks in Bt cotton are directly induced by the Bt toxin. However, some scientists have argued that reduced chemical insecticide use in Bt cotton fields may contribute to non-target pest outbreaks [6], such as *S. exigua* where lower susceptibility to the Bt toxin is substantial [29]. However, reduction of insecticide use in Bt cotton compared to conventional cotton probably is not a factor in releasing *S. exigua* populations in North America, where it is a sporadic, though occasionally devastating, secondary pest of cotton. Instead, outbreaks are often caused or exacerbated by destruction of natural enemies, which normally suppress *S. exigua* populations, through use of conventional insecticides [13], [30]–[32]. Insecticide use has declined even further in North America with the introduction of second-generation Bt cotton varieties expressing two Bt toxins, such as Bollgard II (expressing Cry1Ac and Cry2Ab2) and WideStrike (expressing Cry1Ac and Cry1Fa). The dual-toxin Bt cotton provides increased protection against *S. exigua*, and other lepidopteran pests like *H. zea*, not controlled well by Cry1Ac alone [15], [18], [20], while allowing populations of general predators and parasitoids of *S. exigua* to remain intact.

In China, most transgenic Bt cotton expresses only the single Cry1Ac toxin, and *S. exigua* remains a serious threat to these fields. Although this species is tolerant of Cry1Ac, the toxin is not completely without effect. Several studies have documented the influence of Bt crops expressing Cry1Ac on development, survival and reproduction in *S. exigua* [10], [26], [28], [33], [34]. However, many of the research results are not consistent and even contradictory. For example, Su et al. [10] found no significant negative impact of Bt cotton on *S. exigua* survival curves, pupation rates and pupal weights compared to non-Bt cotton. Likewise, larval mortality was not significantly affected after feeding on Bt cotton, but prolonged larval development time and decreased pupal weight were observed by Arshad and Suhail [28]. In contrast, Nava-Camberos and Ibarra-Frias [33] reported that *S. exigua* growth, survival and fecundity were negatively affected by Bt cotton, while developmental time was not affected. Furthermore, Zhang et al (2007) [34] and Wu et al. (2008) [26] reported that although decreased larval survival, pupal weight and adult fecundity occurred when larvae were fed on transgenic Bt cotton for only one generation, these effects were reduced significantly when larvae fed on transgenic cotton for multiple generations. The conflicting results are probably attributable to the larval food (Bt and non-Bt varieties) used in the experiments, because different concentrations of Cry1Ac toxin are expressed in different varieties [35], or in the same varieties during different growth stages [36], [37]. Therefore, it is desirable to analyze dose-dependent effects of the Bt toxin on *S. exigua* development and reproduction, and consequently on population dynamics.

*S. exigua* is a long-distance migratory species [38]–[43], with the longest flight recorded in the field for any migratory noctuid, 3500 km, and the longest flight distance (179 km) and flight duration (50 h) recorded in laboratory tethered flight tests on flight mills [44]. Large scale migration is one of the most important factors affecting population dynamics and causing outbreaks in immigrant habitat. In North America, long-distance dispersal is responsible for infestations of cotton north of overwintering areas in southern Florida and southern Texas [40], [42], [45], [46]. Several environmental and physiological factors can affect development, reproduction and flight behavior of *S. exigua* [23], [44], [47]–[50], and the sublethal effects of Bt toxin in the larval diet on development, and reproduction may modify flight performance as well.

Herein, we report the novel finding that sublethal effects of low-dose Cry1Ac diet include triggering increased long-flight activity, an effect that may contribute to immigrant population outbreaks. We incorporated different concentrations of pure Cry1Ac protoxin into artificial diet to evaluate the acute dose-dependent effects of Cry1Ac protein on *S. exigua* survival, growth and development, body mass, adult longevity, fecundity and flight performance. In addition, we discuss the implications of our results for non-target migratory insect pest management in Bt crops.

## Results

### Effect of Cry1Ac concentrations in Diet on Growth and Development

**Figure 1:**
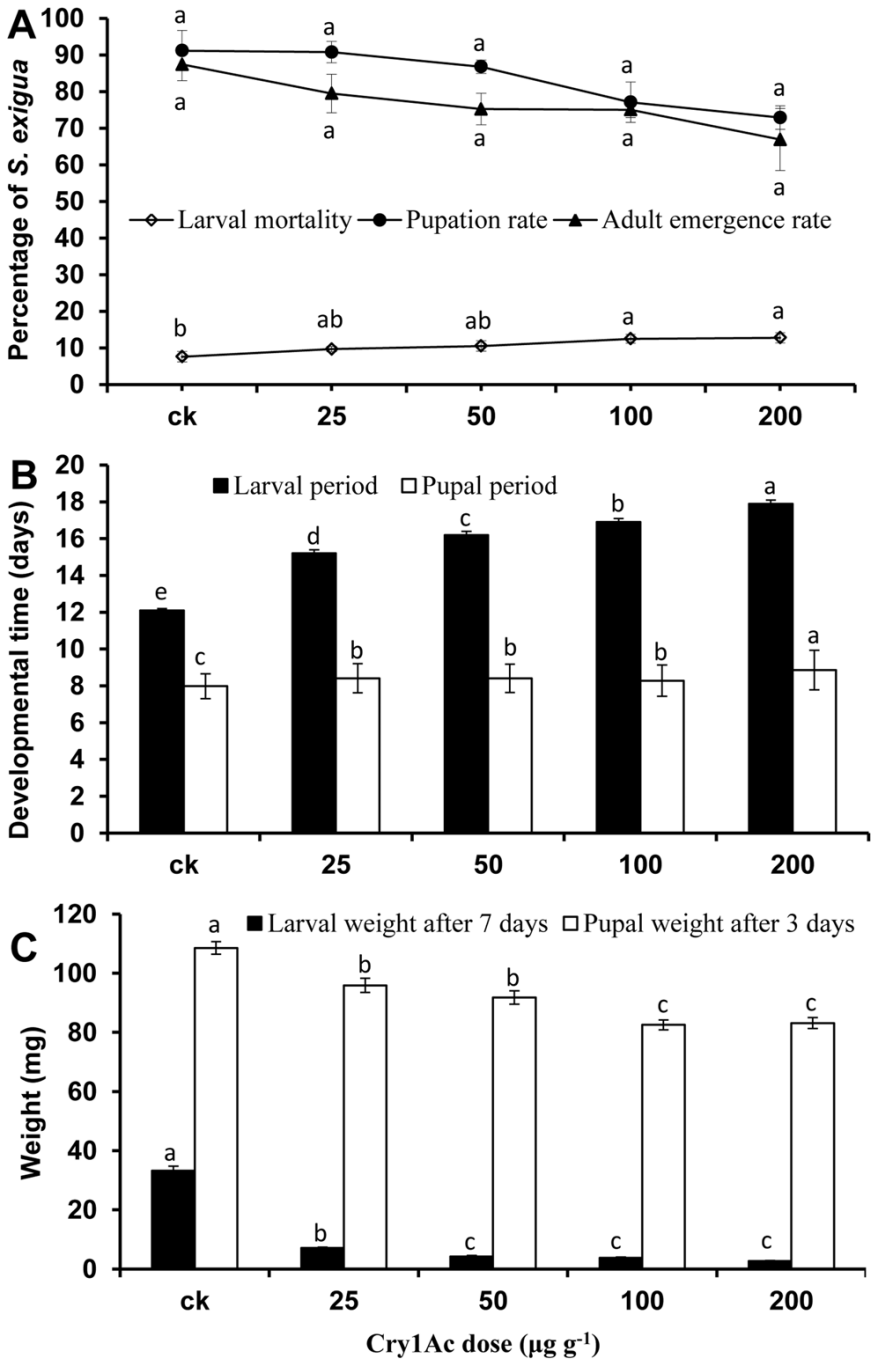
Growth and development of *S. exigua* fed on artificial diet containing different concentrations of Cry1Ac. Non-toxic (ck) artificial diet served as a control. A. Larval mortality, pupation rate, adult emergence rate; B. Developmental time; C. Larval and pupal weight. Data are presented as mean ± SEM. Lines sharing the same letter are not significantly different at 5% level by Tukey’s HSD test.

**Figure 2:**
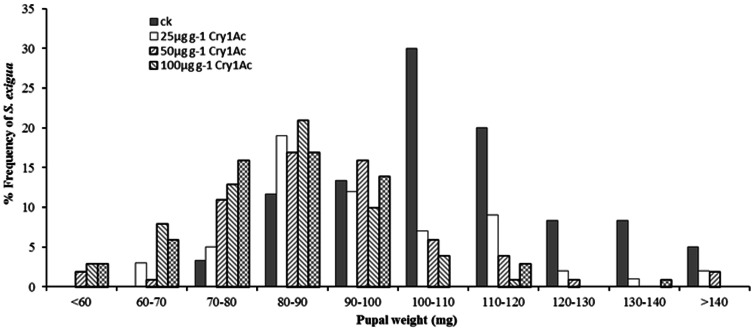
Frequency (%) distribution of pupal weights of *S. exigua* derived from larvae feeding on different concentrations of Cry1Ac. Non-toxic (ck) artificial diet served as a control. Sample sizes were 60 for each treatment and the non-toxic control.

Although Cry1Ac protoxin significantly affected growth and development of *S. exigua* larvae (Fig. 1), mortality of Cry1Ac protoxin fed larvae in the course of the 7 days of the experiment was very low. Larval mortality increased significantly as Cry1Ac protoxin concentration increased (*F*_4,10_ = 6.82, *P* = 0.0136), but the highest only reach 12.81% when fed on a diet containing 200 µg g^−1^ Cry1Ac. No significant differences in larval mortality were observed between larvae fed on the lower concentrations of 25 and 50 µg g^−1^ Cry1Ac protoxin and those fed on protoxin-free control diet (Fig. 1A, *P*>0.05). Subsequently, neither pupation rate (*F*_4,10_ = 3.14, *P* = 0.0645) nor adult emergence rate (*F*_4,10_ = 1.97, *P* = 0.1760) differed significantly between the diets containing Cry1Ac protoxin and the control (Fig. 1A). However, the larval period (*F*_4, 453_ = 191.48, *P*<0.0001) and the pupal period (*F*_4, 372_ = 191.48, *P*<0.0001) were significantly prolonged among those reared on the Cry1Ac protoxin diet compared to the control in a dose-dependent manner (Fig. 1B). Moreover, larval weight after being fed on the Cry1Ac protoxin diet for 7 days (*F*_4, 295_ = 298.44, *P*<0.0001) and pupal weight (*F*_4, 295_ = 26.08, *P*<0.0001) significantly decreased with increasing Cry1Ac concentrations (Fig. 1C). However, a comparison of the frequency distribution of pupal weights between Cry1Ac treatments and control indicates that Cry1Ac protoxin incorporated diet shifted the entire population to a smaller size, rather than causing the loss of a particular subpopulation of heavy pupae (Fig. 2).

### Effect of Cry1Ac Concentrations in Diet on Food Consumption and Utilization

No significant differences were detected among protoxin concentration treatments in RGR (*F*_4, 58_ = 1.701, *P* = 0.162) or RMR (*F*_4, 58_ = 2.807, *P* = 0.054), after fourth-instar larvae were fed on diet for two days (Table 1). However, RCR (*F*_4, 58_ = 2.994, *P* = 0.026) significantly decreased with increasing Cry1Ac protoxin concentrations (Table 1). Despite this dose-dependent decrease of RCR, none of the Cry1Ac protoxin diets treatment showed a significant reduction in ECI (*F*_4, 58_ = 0.749, *P* = 0.563) or ECD (*F*_4, 58_ = 2.395, *P*<0.061) in fourth-instars (Table 1). However, AD was significantly increased in larvae fed on Cry1Ac protoxin diets compared to control larvae (*F*_4, 58_ = 10.601, *P*<0.0001, Table 1).

**Table 1:** Consumption and utilization of artificial diet containing different concentrations Cry1Ac protoxin by fourth instar *S. exigua*.

Cry1Ac concentrations (µg g^−1^)	RGR (mg/mg/d)	RCR (mg/mg/d)	RMR (mg/mg/d)	AD (%)	ECI (%)	ECD (%)
0	1.81±0.18 a	4.44±0.41 a	0.83±0.06 a	0.60±0.03 b	0.40±0.02 a	0.67±0.03 a
25	1.84±0.76 a	4.38±0.42 a	1.21±0.09 a	0.71±0.02 a	0.41±0.02 a	0.58±0.04 a
50	1.74±0.13 a	3.88±0.90 ab	1.08±0.06 a	0.74±0.19 a	0.44±0.02 a	0.61±0.03 a
100	1.22±0.23 a	2.75±0.39 b	0.88±0.08 a	0.78±0.02 a	0.41±0.03 a	0.53±0.05 a
200	1.45±0.26 a	2.52±0.52 b	1.14±0.17 a	0.76±0.02 a	0.39±0.04 a	0.52±0.05 a

Data are presented as mean ± SEM. In each column, data sharing the same letter are not significantly different at 5% level by Tukey’s HSD test. Sample sizes in each column are 12, 12, 15, 13 and 11, respectively.

### Flight Performance of Adults Originated from Larvae Feeding on Cry1Ac

**Figure 3:**
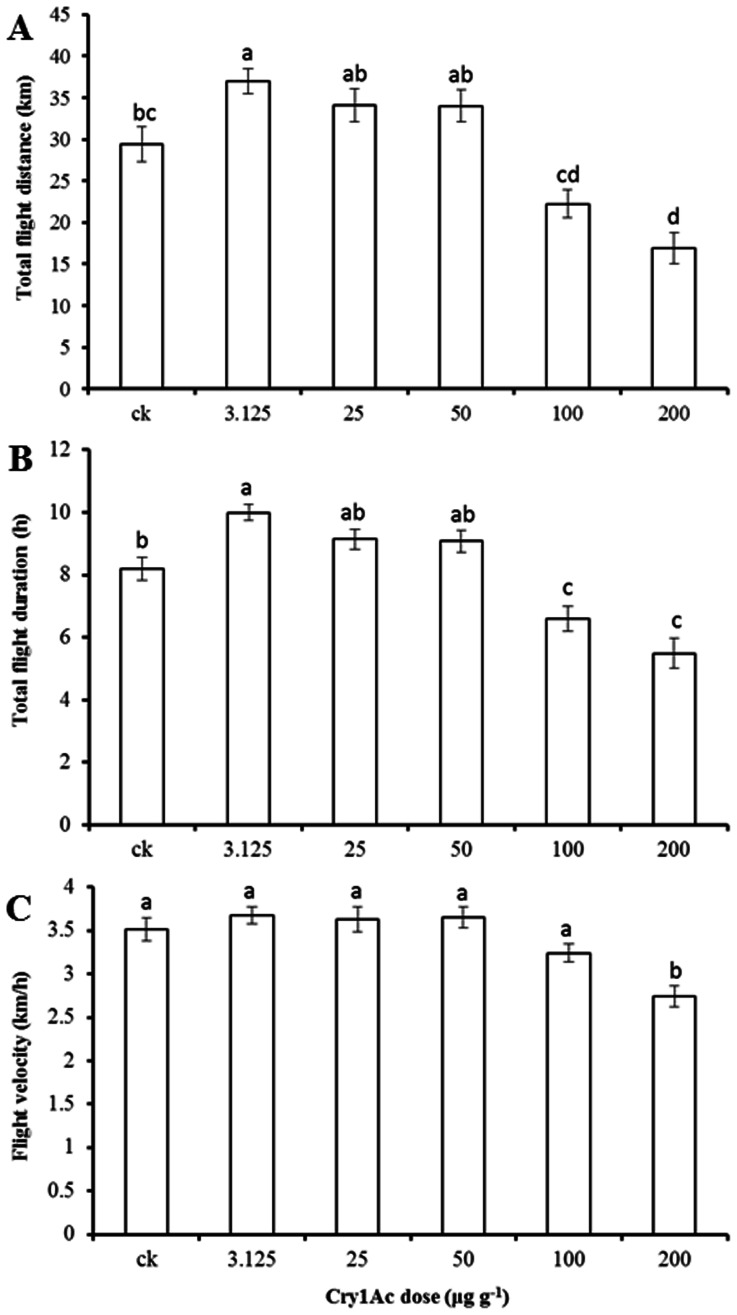
Flight performance of newly emerged *S. exigua* adults derived from larvae feeding on Cry1Ac during a 12-h tethered-flight test. A. Total flight distance; B. Total flight duration; C. Average flight velocity. Sample sizes for non-toxic (ck) and different concentrations of Cry1Ac treatment were 53, 54, 59, 65, 60 and 62, from left to right, respectively. Data are presented as means ± SE. Bars sharing the same letter are not significantly different at 5% level by Tukey’s HSD test.

Significant differences in total flight distance (Fig. 3A, *F*_5, 347_ = 18.148, *P*<0.0001), total flight duration (Fig. 3B, *F*_5, 347_ = 20.907, *P*<0.0001) and flight velocity (Fig. 3C, *F*_5, 347_ = 9.814, *P*<0.0001) of unfed newly-emerged *S. exigua* moths developing from larvae fed on different Cry1Ac diets were observed based on 12 h tethered-flight tests in the laboratory. Flight distance and flight duration of adults developing from larvae reared on diet with the lowest concentration (3.125 µg g^−1^) of Cry1Ac were significantly greater than those of the non-toxic control. Moreover, no significant reductions in flight capacity, including total flight distance, total flight duration and flight velocity, were detected for adults reared from larvae fed on the diets with the three lowest Cry1Ac protoxin concentrations. However, flight duration, distance and velocity of adults reared from larvae feeding on diet with the highest dose (200 µg g^−1^) of Cry1Ac decreased significantly. Flight duration and distance of adults developing from larvae fed diet containing the lowest concentrations (3.125, 25 and 50 µg g^−1^) of Cry1Ac were significantly greater than the two highest concentration treatments (100 and 200 µg g^−1^), although the values for the 25 and 50 µg g^−1^ treatments were not significantly greater than those of non-toxic control (Fig. 3A–B).

**Figure 4:**
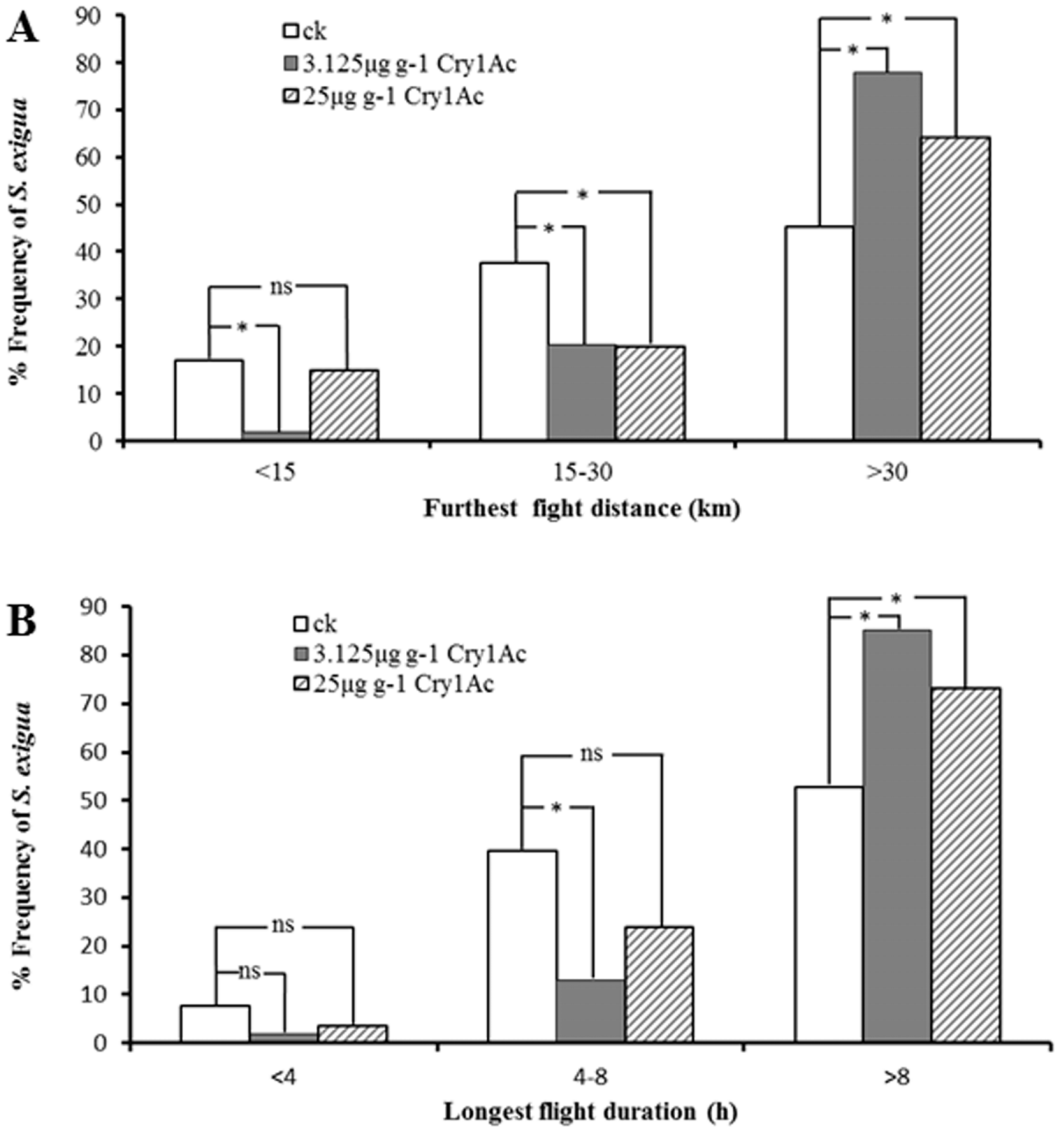
Frequency (%) distribution of the furthest flight distance (A) and longest flight duration (B) of *S. exigua*s during a 12-h flight mill test. Sample sizes for flight treatments of newly emerged adults derived from larvae feeding on non-toxic (ck) and two low-dose of 3.125 µg g^−1^ and 25 µg g^−1^ Cry1Ac incorporated artificial diet were 53, 54 and 59, respectively. Asterisks indicate percentage are significantly different (Chi-Square test, *P<0.05), ns means no significant difference.

Differences between controls and adults reared from larvae of the two lowest Cry1Ac concentration treatments were further analyzed by comparing the frequency distributions of the furthest flight distance and longest flight duration (Fig. 4). A large proportion of adults developing from larvae fed on 3.125 and 25 µg g^−1^ Cry1Ac diet engaged in long-distance flight, with 77.78 and 64.41% making a continuous flight >30 km during the 12-h test period, respectively (Fig. 4A). In contrast, only 45.28% of adults developing from larvae fed on non-toxic diet flew >30 km. Both the differences were significant (*x^2^* = 11.950, *df* = 1, *P* = 0.001 and *x^2^ = *11.122, *df* = 1, *P* = 0.001, respectively). Only 1.9% of adults developing from larvae fed on 3.125 µg g^−1^ Cry1Ac diet did not make a flight of at least 15 km, whereas 16.98% of adults developing from larvae reared on non-toxic diet flew <15 km, and this difference was also significant (*x^2^* = 7.226, *df* = 1, *P* = 0.007). Similarly, *S. exigua* adults developing from larvae reared on the two lowest doses of Cry1Ac diet tended to have significantly more extreme values for the duration of the longest single flight than did those from larvae reared on non-toxic diet (Fig. 4B), with 85.12% (3.125 µg g^−1^ treatment) and 72.88% (25 µg g^−1^ treatment) vs. 52.83% (control) flying >8 h (*x^2^* = 13.128, *df* = 1, *P* = 0.001 and *x^2^ = *4.837, *df* = 1, *P* = 0.001, respectively). Therefore, although adults of all three groups were capable of long-distance and long-duration flights, those developing from larvae fed on a diet containing a low concentration (3.125 and 25 µg g^−1^) of Cry1Ac displayed a significant increase in the percentage of long fliers on the flight mill system.

Parameters of adult flight performance across controls were regressed on pupal weight. A significant relationship was found, but it was quadratic, not linear: Total flight distance (y = −0.0218x^2^+4.6852x−211.46, R^2^ = 0.592, *F*_2,50_ = 36.271, *P*<0.0001), flight velocity (y = −0.001x^2^+0.2212x−6.683, R^2^ = 0.456, *F*_2,50_ = 20.916, *P*<0.0001), flight duration (y = −0.0042x^2^+0.9123x−39.322, R^2^ = 0.725, *F*_2,50_ = 66.010, *P*<0.0001), indicating that adults developing from middle-weight pupae exhibited stronger flight capacity.

### Reproduction of Adults Originated from Larvae Feeding on Cry1Ac

No significant differences were observed in the pre-oviposition period (*F*_4,152_ = 0.591, *P* = 0.670), oviposition period (*F*_4,152_ = 0.668, *P* = 0.615), mating percentage (x^2^ = 3.526, *df* = 4, *P* = 0.474) and frequency (*F*_4,152_ = 0.295, *P* = 0.881), and the longevity (female, *F*_4,152_ = 1.239, *P* = 0.297; male, *F*_4,152_ = 1.343, *P* = 0.257) among adults reared from larvae feeding on different Cry1Ac protoxin concentration treatments (Table 2). However, the lifetime number of eggs laid per female (*F*_4,152_ = 12.389, *P*<0.0001) was significantly reduced in adults reared from larvae feeding on Cry1Ac protoxin compared to the controls, regardless of the experimental Cry1Ac concentrations (Table 2). It is possible that reduced size caused by rearing larvae on diet containing Cry1Ac led to the observed reduction in lifetime fecundity [51] To examine this possibility, the lifetime number of eggs laid was regressed on pupal weight, revealing a significant, positive linear relationship (y = 10.975x − 351.41, R^2^ = 0.8226, *F*_1, 58_ = 268.987, *P*<0.01).

**Table 2:** Reproductive performance of *S. exigua* adults reared from larvae feeding on diet containing different concentrations of Cry1Ac protoxin.

Cry1Ac concentrations (µg g^−1^)	Samples (n)	Pre-oviposition period (days)	Oviposition period (days)	Total no. eggs laid in lifetime	Mating percentage (%)	Mating frequency (Spermatophores per female )	Longevity (days)	
							Female	Male
ck	30	2.13±0.08 a	6.73±0.49 a	842.00±52.95 a	100.00	3.17±0.21 a	9.37±0.71 a	9.77±0.64 a
25	32	2.34±0.14 a	6.94±0.40 a	577.47±39.95 b	96.88	3.09±0.20 a	10.19±0.62 a	11.22±0.65 a
50	30	2.30±0.10 a	7.43±0.51 a	544.50±37.16 b	96.67	3.03±0.22 a	10.70±0.60 a	11.37±0.58 a
100	33	2.24±0.09 a	6.73±0.45 a	514.30±25.59 b	90.91	2.89±0.17 a	9.27±0.48 a	10.21±0.57 a
200	32	2.25±0.05 a	6.41±0.45 a	498.28±39.36 b	93.75	3.09±0.21 a	9.16±0.59 a	9.91±0.74 a

Data are presented as mean ± SEM. In each column, data sharing the same letter are not significantly different at 5% level by Tukey’s HSD test.

## Discussion

### Lower Susceptibility to Cry1Ac may Contribute to *S. exigua* Outbreaks in Transgenic Bt Crops

No significant direct negative effects of Cry1Ac toxin on *S. exigua*, including larval mortality, pupation rate and adult emergence rate were detected when larvae were fed diet incorporating the lower Cry1Ac doses of 25 to 50 µg g^−1^ in our experiments, confirming low susceptibility to Cry1Ac toxin in this species. However, significant negative sublethal effects were observed in larval developmental time and pupal weight. These results are consistent with those of previous studies comparing larvae fed on Bt crops expressing the Cry1Ac endotoxin compared to non-Bt crops [15], [28], [35]. In contrast, Nava-Camberas and Ibarra-Frias (2000) reported direct adverse effects of Cry1Ac toxin on *S. exigua*, where growth and survival were negatively affected by the Bt toxin contained in transgenic NuCOTN35B cotton while developmental time was not affected [33]. The differing results may have been caused by different Cry1Ac toxin concentration in cotton varieties or tissue [35], [37], [52]. Our results indicate that *S. exigua* larval mortality, larval period, larval weight, pupal period and pupal weight changed in a Cry1Ac dose-dependent manner.

The sublethal effects we observed in *S. exigua* of delayed development and reduced body size may be explained by a decrease in food consumption. The significant reduction in RCR observed in fourth instars may have been caused by intoxication or reduced palatability of the diet containing Cry1Ac. Interestingly, AD did not decrease with increasing Cry1Ac concentrations in the diet, but instead was significantly greater in all Cry1Ac treatments compared to the control. This result suggests that *S. exigua* is well-adapted to digest food despite containing Cry1Ac. Furthermore, RGR, RMR, ECI and ECD were not significantly reduced in the presence of Cry1Ac regardless of concentrations. This ability of *S. exigua* to consume and utilize diet containing Cry1Ac protoxin is not comparable to that of insect pests susceptible to Cry1Ac [53]. The increase in larval development time after feeding on diet containing Cry1Ac might result in an increase in the number of larval instars. Previously, we found that poorer quality larval food led to a greater than normal number of instars in *S. exigua* [47], a phenomenon also found in other insects [54].

### Increased Flight Distance and Duration Triggered by Low dose Cry1Ac

No significant differences were detected in adult reproductive attributes, including pre-oviposition period, oviposition period, mating percentage and frequency, and longevity, except that lifetime fecundity decreased significantly when larvae developed on diet containing Cry1Ac. There was a significant reduction in pupal weight when larvae were reared on Cry1Ac-containing diet, which may result in reduction in adult lifetime fecundity. We found that fecundity increases with pupal weight. Likewise, Tisdale and Sappington (2001) showed a strong correlation between *S. exigua* pupal weight and fecundity, which can be enhanced by carbohydrate in the adult diet [51]. This kind of relationship is consistent with that observed in the oriental armyworm, *Mythimna separata*, as well [55].

Larval diet can have a large effect on *S. exigua* pupal weight [56], [57], and pupal weight is positively related to lifetime fecundity (Table 2) [51]. Thus, it is possible that the observed reduction in fecundity in our experiments is a direct result of poor development on diets that are suboptimal because of their Cry1Ac content. The full story seems not so simple, however. Our data also indicate that there is no problem digesting and utilizing the diet laced with Cry1Ac, despite a lower consumption rate of diet containing higher concentrations. Weight of pupae from larvae fed diet with lower concentrations of Cry1Ac was intermediate between control pupae and those fed diet with higher concentrations. But the adults from these intermediate-weight pupae flew further and for longer duration than did the larger control moths reared on non-toxin diet. Thus, it is possible that a developmental pathway for enhanced migratory behavior was triggered by the signal of a suboptimal larval diet. As Cry1Ac concentration increased, the physiological effects perhaps overwhelmed the signaling effects so that the lesser energy reserves or lesser physical robustness of the adult compromised its flight capacity.

Insect migration is an important behavioral adaptation for spatially tracking ephemeral, seasonal habitats [58], [59]. In the case of facultative migration, environmental cues trigger or inhibit the onset of migration, and these cues can be associated with habitat quality [60]–[62]. For example, larval food may not only directly influence biological parameters of migratory insects such as growth, survival, and development rates, but may also serve as a cue at developmental decision points for determining alternative migration and reproduction life history pathways. In previous studies, we found that different larval host plants of *S. exigua* significantly affected adult flight performance [47]. Similarly, we found that in *M. separata* a certain extent of starvation during the larval stage significantly increases the proportion of migrant adults, and that migrants lay fewer eggs [63].

We hypothesize that the reduced adult lifetime fecundity observed in *S. exigua* ingesting diet containing low doses of Cry1Ac is a direct effect of suboptimal diet on size and energy reserves of the adult. But in addition, a physiological response to a perceived poor diet triggers enhancement of migratory flight potential. In other words, the suboptimal larval diet triggers a facultative adaptive life history strategy in the adults to escape the poor quality natal location to find a new, potentially more-appropriate habitat by migration. A direct effect of Cry1Ac on reduced fecundity, rather than reflecting a facultative diversion of resources from reproduction to flight, is suggested by previous experiments that show there are no such tradeoffs between reproduction and flight in this species [23]. If this hypothesis is correct, the infestation of Bt cotton by *S. exigua*, no longer suppressed indirectly by conventional insecticides that used to be applied to control the target pests of Cry1Ac, may lead to more intense outbreaks in the north by immigrants. This is because increased long-flight activity, triggered as a sublethal effect of Cry1Ac ingestion in the south, may result in increased numbers dispersing to the north.

Our results suggest a possible connection between increased long-flight activity of *S. exigua* adults and larval feeding on diet incorporating a concentration of Cry1Ac (3.125 µg g^−1^) close to that expressed in Bt cotton tissues [18], [64]. The observed increase in long-flight activity may reflect a change in migratory tendency and distance. By themselves, however, these data are inadequate to firmly draw the conclusion that feeding on Bt cotton increases migratory behavior in the field. Secondary plant compounds can vary from one cultivar to another, and might have a critical effect on larval fitness and subsequent migratory behavior and reproduction of adults. For example, gossypol levels can vary greatly from one cultivar to another, and protects the cotton plant from many species of insect. Gossypol affects developmental parameters of *H. zea* and *H. virescens* [65], [66]. Likewise, *S. exigua* growth, development, food utilization and adult reproduction were significantly influenced by different levels of gossypol in cotton [67], and it is reasonable to hypothesize that it might directly or indirectly affect flight capacity as well. In addition, differences in the Cry1Ac toxin concentration and form (protoxin versus toxin) between artificial diet and Bt cotton tissues may also have an impact on sublethal effects and migratory behavior. The next step in testing the hypothesis that migratory behavior of *S. exigua* adults increases when larvae are exposed to Cry1Ac will be to examine the response of larvae reared on Bt cotton tissues instead of artificial diet.

Finally, although adult reproduction was not investigated in the treatment where larvae were fed diet at the lowest concentration of 3.125 µg g^−1^ Cry1Ac, all other Bt concentrations tested resulted in a significant reduction in overall eggs laid. Therefore, it is possible that egg laying fitness costs in the dispersing moths may reduce their pestiferous potential after emigration to distant fields. However, exposure of brown planthoppers to a chemical insecticide not only induced enhanced flight capacity [68] but also increased the number of eggs laid [69]. Reproductive timing and capacity are not correlated with facultative migratory behavior and physiology in *S. exigua* [23], so the effects of field-relevant doses of Cry1Ac on realized fecundity remain to be determined experimentally.

## Materials and Methods

### Insects

*S. exigua* used in the experiments were from a colony that originated from a population collected near Beijing, China (115.58°E, 40.18°N). The colony had been maintained for 55 generations when the experiments began. Larvae were reared on a soybean-corn flour artificial diet developed for *S. exigua* by our research group and as described previously [47] at a constant temperature of 28±1°C, 60–70% RH and photoperiod of 14 L:10D (light period was from 07∶00 to 21∶00). Pupae were sexed on the third day and adult males and females were allowed to emerge in separate transparent containers. Adults were transferred in pairs to 850-mL jars provided with wax paper for oviposition, and eggs were collected daily. Jars were covered with gauze to facilitate ventilation and adults were provided with 5% honey solution (v/v) replaced daily.

### Cry1Ac Protoxin and Diet Preparation

Cry1Ac protoxin was purchased from Zhongbao Science and Technology Co. (Institute of Plant Protection, Chinese Academy of Agricultural Science). Cry1Ac protoxin was produced from a strain of *B. thuringiensis ssp*. *Kurstaki* according to the methods of Luo et al. (1999) and Pan et al. (2010) [70], [71]. The Cry1Ac protoxin showed as a clear band at about 130 kDa in SDS-PAGE. The purified Cry1Ac protoxin was lyophilized and stored at −70°C, and dissolved in double distilled water before use. During the above artificial diet preparation, dissolved Cry1Ac protoxin was serially added to the liquid artificial diet before solidification, and mixed in a blender for about 60 s. The final Cry1Ac concentrations of 25, 50, 100, and 200 µg g^−1^ diet were examined for effects on larval development and subsequent adult reproduction. However, an additional lower Cry1Ac concentration of 3.125 µg g^−1^ diet, which closely resembles the concentration in Bt cotton tissues [18], [64], was tested for subsequent effects on adult flight. Control insects were reared on artificial diet without Cry1Ac protoxin.

### Growth and Development of Larvae Feeding on Cry1Ac

Newly hatched larvae (≤12 h after hatch) of *S. exigua* were reared individually in wells of a 24-well plate (20 ml capacity). Approximately 0.2 g fresh diet was transferred to each well, and the larvae were provided with fresh diet every 3 days until they either died or pupated. There were 48 larvae for each replicate and the experiment was replicated three times for a total of 144 larvae per treatment. Larval mortality, larval weight on the 7th day after initiating the experiment and duration of the larval period were recorded. After pupation, sixty 3-day-old pupae were randomly selected to weigh, and pupation rate, pupal period and adult emergence rate (%) were all recorded. Larvae were considered dead if they were unable to move in a coordinated manner when prodded with a blunt probe.

### Food Consumption and Utilization of Larvae Feeding on Cry1Ac

Because *S. exigua* larvae feed voraciously as 4th instars, a single, pre-weighed, 4th instar larva that had been starved for 12 h was released in each well in the plate. Cubes of artificial diet containing 0 (control), 25, 50, 100, and 200 µg g^−1^ Cry1Ac protoxin were placed singly in each well after recording their fresh weight. After each day of larval feeding, the weights of larvae, uneaten diet and larval frass were recorded. Samples for each diet concentration treatment included at least 15 larvae. A blank control diet without larva was used to correct for diet weight change from water loss, according to the method of Chen et al. (1987) [72]. Indices of food consumption and utilization, including relative growth rate (RGR, g/g·d), relative consumption rate (RCR, g/g·d), relative metabolic rate (RMR, g/g·d), approximate digestibility (AD, %), efficiency of conversion of ingested food into body matter (ECI, %), and efficiency of conversion of digested food into body matter (ECD, %), were calculated as proposed by Waldbauer (1968) [73], and as conducted by Chen et al. (1987) [72] and Wu et al. (2008) [26].

### Reproduction of Adults Emerged from Larvae Feeding on Cry1Ac

About 35 pairs of adults that emerged from larvae fed on diet containing different Cry1Ac concentrations were randomly selected, as were another 30 pairs of adults reared from control larvae. Egg output was recorded daily, and food changed until the adults were dead. At death, the female was dissected and the number of spermatophores in the bursa was determined. This revealed whether a pair was mated and the number of matings per pair, and allowed calculation of the mating percentage and other reproductive parameters. The pre-oviposition period (POP), lifetime fecundity, mating frequency, and mating percentage were used to evaluate changes in reproduction in response to different Cry1Ac protoxin concentration treatments. These parameters were determined following the methods employed in previous studies [23].

### Flight Performance of Adults Emerged from Larvae Feeding on Cry1Ac

Flight tests of tethered moths were conducted on a 32-channel flight mill system, as described in previous studies [23], [44], [47]–[49]. Newly-emerged *S. exigua* adults without supplementary nutrition and reared from larvae feeding on diets of different Cry1Ac concentration were randomly selected for 12 h of tethered flight on flight mills. Such unfed moths reflect the relationship between flight capacity and larval food condition [47]. The flight tests were performed in a climate chamber. Temperature and humidity in the flight room were maintained at 24±1°C and 70% ±10% RH, respectively, conditions promoting maximum flight capacity of *S. exigua* [49]. Data logging (software supported by Jiaduo Co. Henan China) began at 20∶00 h in the evening and terminated at 08∶00 h the next morning under dark conditions. Flight parameters, including total flight distance and duration, average velocity, furthest flight distance and longest flight duration were automatically recorded by the system. Longest flight duration and furthest flight distance were those of the single longest uninterrupted flight. Approximately 60 adults were observed for each treatment. Data were excluded from the analyses if the adult was detached from the tether or broke a wing during the test period. In all treatments, flight data were pooled across genders as there is no significant difference in the migratory flight capacity or behavior of males and females [23], [44].

### Data Analysis

All data obtained from the studies are presented as means ± SEM. Treatment effects on variables were evaluated by one-way analysis of variance (ANOVA). If the ANOVA indicated a significant difference, the means responsible for that difference were identified using Tukey’s HSD test. All the percentage data were arcsine transformed before ANOVA to meet the assumptions of normality. Differences in mating percentage between the treatments were compared by Chi-squared tests. Differences in flight parameters between moths reared from larvae in the 3.125 and 25 µg g^−1^ Cry1Ac treatments and from unexposed controls were further analyzed by comparing the frequency distribution of longest flight duration and furthest flight distance by Chi-squared tests. All statistical procedures were performed with SPSS software (SPSS 17.0).
